# The Present Condition of the Dental Profession

**Published:** 1908-05-30

**Authors:** 


### Dental Department.

### THE PRESENT CONDITION OF THE DENTAL PROFESSION.

Two cases of considerable interest have recently come into the law courts; one in which a skilful dentist, practising in the West End of London, endeavoured to recover a fee of £580 for professional services rendered, the other in which an unqualified dentist was charged before the coroner's court with causing the death of a patient, by the injection of a toxic dose of cocaine; in the first case the dentist recovered about half his fee, or over £250; in the second, the unqualified man received the mildest of censures. These two extreme cases draw marked attention to the very peculiar position which dentistry occupies at the present day.

Dentistry, an adopted daughter of Medicine, is comparatively young; just thirty years ago, in 1878, the Dental Act was passed, which required that all those who were engaged in the profession of dentistry, all those who combined with their regular avocation the casual practice of dentistry, and all those who were assistants to, articled or apprenticed to dentists, should register their names on a dental register, to be kept at the offices of the General Medical Council, and that none other should take the style and title of dentist, unless properly trained at recognised schools, and examined and passed by legally appointed bodies.

The result of this Act was that all those who were genuinely in practice as dentists became legally entitled to style themselves dentists, and hundreds of others with very little or no training, chemists, jewellers, mechanics, etc., established themselves as registered dentists in like manner; there also sprang up dental schools, attached more or less to general hospitals, where students were trained according to the requirements of the Act, and received the instruction necessary to pass the examinations of the various boards. For the first few years the registered men far outnumbered the qualified, and had matters very much their own way; they practically received a monopoly from the State, and a most lucrative thing they found it.

The Dental Act, of 1878, originated with the medical profession, and the framers of it had in their minds the making of dentistry as much a special branch of medicine as rhinology, laryngology, etc., and they thought that the syllabus required for the license in dental surgery might, in time, be so broadened, as to include a general medical education, and lead up to all dentists taking the M.R.C.S., L.R.C.P., plus their special dental subjects.

However, as time passed, it began to be apparent that matters were not going on according to the hopes of the framers of the Act; on the contrary, either because dentistry, *per se*, had no attraction for those who could afford the time and money for the necessary training, or because they thought the game not worth the candle, candidates did not come forward in sufficient numbers to fill the vacancies on the register caused by the death or retirement of the older registered men, let alone in sufficient numbers to cope with the increase in population; and, moreover, those men who were induced to take the general medical course were, either by the greater glamour of medicine, or by the higher social standing which a medical man takes in the eyes of the public, led to forsake dentistry and went over entirely to the practice of medicine. Later on, worse troubles arose out of this unfortunate Act, for there is a law of supply and demand, against which it is useless to legislate.

The moneyed classes, and upper social strata of society naturally went to the hospital-trained man, the registered man received his support from the middle classes, and there was left a great mass of the public, the lower middle, and lower classes, without any one to minister to their dental wants. Such a condition of affairs has never, as far as we are aware, arisen in regard to medicine; the supply of doctors in relation to the population has always been equal to its requirements, or even a little in excess; there have always been the large general hospitals and the provincial infirmaries, where the poorer classes could be sure of efficient and adequate medical treatment.

The dental department of general hospitals and infirmaries, and the special dental hospitals, were, and are, totally inadequate to meet the requirements of the poorer public, nor can they ever be made adequate. The most these public institutions could and can do, is to relieve pain by the extraction of the offending tooth. The question might be raised—what of the special dental hospitals and schools? All these do is to choose suitable patients in numbers sufficient to give their students the necessary amount of training and experience; the number of patients they can treat is infinitesimal, compared with the vast bulk of the populace. Therefore, the supply not being equal to the demand, there began to practise men who had no qualifications and no training—the "unqualified"; their numbers were few at first, and the General Medical Council made desperate efforts to stop them. Prosecutions were instituted again and again, but only where these men styled themselves "dentists," were the Council able to obtain convictions, for according to the wording of the Act, it is only illegal to take the title of dentist; anyone may practise dentistry to his heart's content.

After these unsuccessful prosecutions, the number of unqualified men practising increased by leaps and bounds, so that to-day their name is legion. Companies were formed with branches all over the Kingdom, mostly carried on by untrained and unqualified men; they flourished and were successful and attracted capital, and to-day their position is a strong one.

Companies are run solely for making money; where a professional man practises in a district, there is a certain definite standard of conduct which it is necessary for him to maintain, and he has to stand by his ability and work; for by these his brother practitioners and his patients judge him. His fees also have to be such as are reasonable to the district. Not so with companies; a branch is started in a district, the district is flooded with pamphlets, and glowing advertisements appear in the local papers, an assistant is placed in charge and told he must make it pay; his remuneration is arranged according to the amount of profit he can make, the class of work he does is immaterial, the eyes of the directors are ever on the balance sheet, and if the branch does not pay, what is more easy than to shut it up and start elsewhere, the successful branches paying for the unsuccessful ones.

Now this is bad for the public and bad for the dental profession, dragging it down to the level of a trade, and making those men of better education and standing still less desirous of entering a calling set about by so many deplorable conditions. Thus it has come about that dentistry has reached this present climax, that hundreds and hundreds of men are practising on their fellow creatures operations which, to be done properly, require the most delicate judgment and skill, and that amount of accumulative clinical knowledge which, as in medicine, has been built up by years of experience, and which can be acquired only at special hospitals and schools, and from men who themselves have been before in like manner taught.

Every day scores of people are receiving anæsthetics, local and general, at the hands of the unqualified, and needless to say there have been many fatalities. Inquests have been held, and the result has invariably been that, as in the case referred to above, the unqualified man has received a mild censure from the coroner or chairman of Quarter Sessions, and has been quite free to go on again, having gained a little experience at the cost of a patient's life.

The profession suffered another invasion of a very different character from that of the unqualified man; this was an influx of many dentists of high technical skill from America, where dentistry was much in advance of that in England. By reason of their practising a form of dentistry which appealed to the wealthy public, namely, bridgework, and by the *éclat* which American dentistry obtained in contrast with the slower and more conservative methods of the English dentists, they were able to command amazing fees.

There is another factor in the situation, and that is, though we are years behind America and some of our Colonies, and, indeed, of many continental nations, in the amount of importance we attach to the care of our teeth, still the mass of the people is slowly waking up to the supreme importance of having the teeth properly cared for; moreover, there is just coming into force an Act for the compulsory medical inspection of children attending the elementary schools, which will include the inspection of the mouth and teeth. Judging from statistics which have already been collected, it is probable that at least 87 per cent. of children will be found to have defective teeth; inspection without treatment could be nothing but a farce, and presumably the Act intends to lead up to the treatment of the various defects from which the children may be found to suffer.

That branch of dentistry which deals with the treatment of children's teeth, more than any other, requires special knowledge and skill, and the unqualified man is totally incapable of dealing with it. The unqualified man's dentistry consists at the best, of the extraction of teeth and their substitution by means of plates, and when the appalling defects in the teeth of children in elementary schools are once realised, a very difficult problem will await solution, a vast amount of work requiring to be done and no one with sufficient training and experience to do it. The case is different from medical inspection and treatment, for a medical man can diagnose and prescribe for a considerable number of children in an hour, but to attend properly to a child's teeth, even a child with moderately good teeth, would probably take an hour per child. The State treatment of children suffering from disease cannot but be fraught with enormous difficulties, but if it is to include dental disease (without which it is practically a waste of time) under present conditions, the difficulty seems well nigh insuperable.

The dental profession and some small number of medical men have not been unobservant of the approach of this climax: many meetings have been held and many sugestions made, but a solution seems far off. The question is why do not men of sufficient means and education take up dentistry? it is a profession and it is lucrative. One hears so much of the overcrowding of the professions, and the cry is so often raised "what shall we do with our boys?" The line of reasoning taken by parents and guardians, seems to be, that if the boy has to spend four years in professional studies, has to pass so many examinations akin to medicine, and at about the same cost, why not make him a doctor straight away? They overlook the fact that the medical profession is already overcrowded. The dental profession, on the other hand, is the least overcrowded of all professions, for if all unqualified men were to be prevented from practising, the qualified men would be totally unable to cope with the work. The interesting question arises as to whether it is possible by means of legislature, to graft a new profession on to an established society; a somewhat similar experiment is about to be tried in the new Midwives Bill, and the result cannot but be watched with interest.

It has been suggested that the present standard of qualification should be lowered, and the pathway of candidates made easier, but no Examining Board is likely to do this—indeed, the tendency is all the other way. Knowledge does not move backwards, and as time goes on, more preparation and higher attainments are required in every walk of life, and while medical knowledge is advancing by leaps and bounds, it would be a deplorable thing for dentistry to take a retrograde step, or even to stand still.

Those who are interested in dentistry, the medical profession especially—for they are every day increasingly availing themselves of the dentists' aid to the great benefit of their patients—can only hope that as time goes on the right sort of men will come forward in sufficient numbers to undertake the work waiting to be done. Then the unqualified dentists and the artificial tooth companies will retire into that oblivion from which circumstances have called them.

